# A Correlation of Permanent Anterior Tooth Fracture with Type of Occlusion and Craniofacial Morphology

**DOI:** 10.5005/jp-journals-10005-1194

**Published:** 2013-08-26

**Authors:** Renu Chaturvedi, Ashish Kumar, Vivek Rana, Abhai Aggarwal, Lokesh Chandra

**Affiliations:** Reader, Department of Pedodontics and Preventive Dentistry, IDST Dental College, Modi Nagar, Uttar Pradesh, India, e-mail: dagur_ashish@yahoo.com; Senior Lecturer, Department of Oral Surgery, Kalka Dental College Meerut, Uttar Pradesh, India; Professor, Department of Pedodontics and Preventive Dentistry Subharti Dental College, Meerut, Uttar Pradesh, India; Professor, Department of Pedodontics, Kalka Dental College, Meerut Uttar Pradesh, India; Reader, ITS Dental College, Murad Nagar, Ghaziabad, Uttar Pradesh India

**Keywords:** Tooth fracture, Craniofacial morphology, Occlusion

## Abstract

**Aims:** To assess the relationship of anterior tooth fractures with type of occlusion and craniofacial morphology.

**Materials and methods:** The study was conducted on 76 subjects of age group 9 to 13 years with at least one fractured permanent anterior teeth. Lateral cephalograms were taken and study models were prepared for each subject with prior consent of their parents. Then cephalometric tracings were done and overjet was recorded through study models.

**Statistical analysis used:** Standard error of mean (SEM) and unpaired t-test has been applied to test the significant difference between the seven parameters under consideration. Karl Pearson correlation test has also been used to correlate all the parameters used in this study with each other. All the tests were performed at 5 and 1% levels of significance.

**Results:** Frequency of tooth fracture increases with increasing overjet. At 5% level of significance, significant difference were observed between the standard values and observed values for overjet measurement, SNA angle, SNB angle, ANB angle, upper incisor to NA (angle), upper incisor to NA (linear) and interincisal angle for overall data and also for both male and female data separately.

**Conclusion:** Probability of permanent anterior tooth fracture increases with increasing overjet. A significant difference was observed between the standard value and the observed values of all parameters under consideration.

**How to cite this article:** Chaturvedi R, Kumar A, Rana V, Aggarwal A, Chandra L. A Correlation of Permanent Anterior Tooth Fracture with Type of Occlusion and Craniofacial Morphology. Int J Clin Pediatr Dent 2013;6(2):80-84.

## INTRODUCTION

Anterior teeth have a great impact on an individual's personality as they play a critical role in the speech, esthetics and masticatory functions of an individual.^1^ Identification and understanding of the risk factors is helpful in diagnosing and in preventing cases more prone to anterior tooth fracture.^2^ The aim of the foregoing study is to evaluate the relationship of anterior tooth fracture with type of occlusion and craniofacial morphology so that the results drawn from the study can be further applied in preventing anterior tooth fractures.

## SUBJECTS AND METHODS

One thousand patients were examined and out of them 76 patients were selected for the study. Lateral cephalograms and study models were made for each patient.

The criteria for selecting the patients were that age should be in between 9 and 13 years with at least one noncarious fractured anterior tooth. The fractured tooth in each patient should not be susceptible to fracture due to presence of any developmental defects and first permanent molars should be present for assessment of occlusion.

## METHODOLOGY

**Fig. 1:**
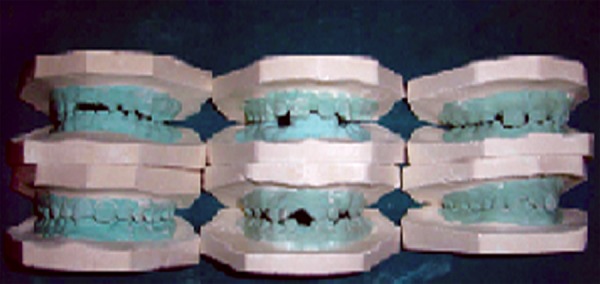
The study models of patients chosen for the study to check the occlusion and overjet

Impressions of upper and lower jaws of each patient were made using irreversible hydrocolloid material. Impressions were poured in dental stone type III and study models were prepared which were used to check the occlusion and overjet of the patient (Fig. 1).^3^

**Fig. 2:**
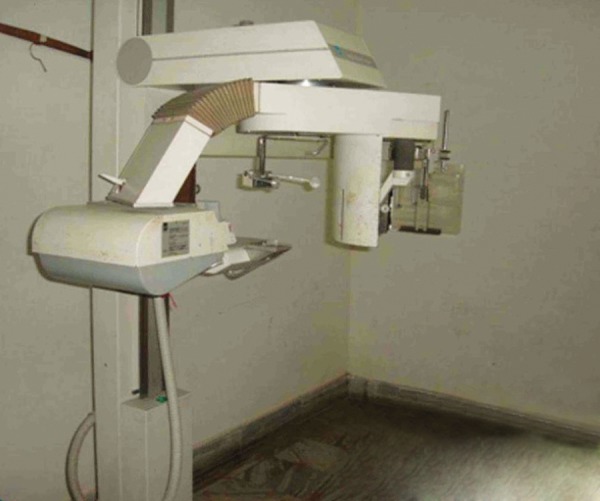
The cephalostat machine used for taking the lateral cephalograms of the patients

**Fig. 3:**
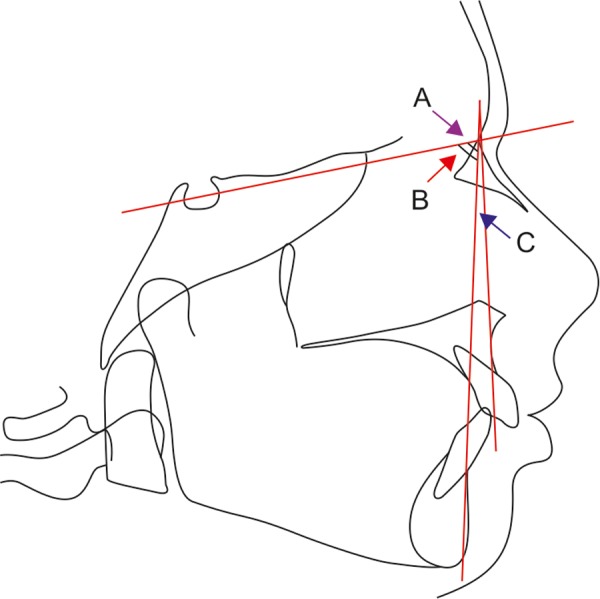
The lateral cephalometric tracings done on patient's lateral cephalograms. Here, A: SNA angle; B: SNB angle; C: ANB angle

Lateral cephalograms of all the patients were taken by cephalostat machine (Villa Sistemi Medicali, model MR 05 Type 84086500) using 20.3 × 25.4 cm/ ‘8’ × ‘10’ inches film cassettes equipped with Kodak film and intensifying screens (Fig. 2). Cephalograms were studied for the cephalometric landmarks and various planes and angles were traced on an acetate matte tracing paper (0.003 inch thick, 8 × 10 inches) using sharp 3H drawing pencil (Fig. 3).

**Fig. 4:**
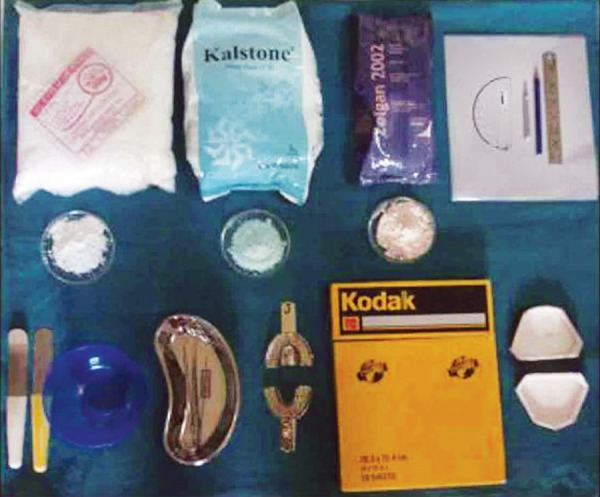
The materials used for this study

Following materials were used for the study (Fig. 4):

1. Kodak film 20.3 × 25.4 cm/ ‘8’ × ‘10’ inches.
2. Irreversible hydrocolloid material (Zelgan 2002, Dentsply dust-free Alginate).
3. Dental stone type-III (Kalstone, Kalabhai Karson, Mumbai).
4. Plaster of Paris.
5. Stainless steel perforated stock trays
6. Straight plaster spatula
7. Curved plaster spatula
8. Rubber bowl
9. Mouth mirror
10. Dental probe
11. Tweezer
12. Metallic scale
13. Divider
14. Protractor
15. 3H lead pencil
16. Lead acetate matte tracing paper.

## RESULTS

The data were tabulated in the following tables.

- *Table 1:* Males are more prone to fracture than females.
- *Table 2:* The probability of tooth fracture increases with increasing age.
- *Table 3:* Commonest tooth to get fractured is right upper central incisor.
- *Table 4:* The frequency of individual tooth fracture increased with increasing overjet.
- *Table 5:* The Z-test was applied to test the significant difference between standard value and sample observation, a significant difference was observed for overjet measurement, SNA angle, SNB angle, ANB angle, upper incisor to NA (angle), upper incisor to NA (linear) and interincisal angle at 5% level of significance.
- *Tables 6A and B:* At 5% level of significance, a significant difference was found for all the parameters under consideration *viz* overjet measurement, SNA angle, SNB angle, ANB angle, upper incisor to NA (angle), upper incisor to NA (linear) and interincisal angle for male and female data respectively.
- *Tables 7A and B:* Using unpaired t-test, no significant difference was found at 5% level of significance, i.e. p > 0.05. However, 95% confidence limits are shown for all the parameters *viz* overjet measurement, SNA angle, SNB angle, ANB angle, upper incisor to NA (angle), upper incisor to NA (linear) and interincisal angle.
- *Table 8:* Significant and strong positive correlation at 0.001 level of significance between SNA angle and overjet measurement and an inverse correlation was found between SNB angle and overjet measurement.

**Table 1:** **Table 1:** Sex distribution of the study sample

	*Frequency*	*Percentage*
Males	46	60.52
Females	30	39.48
Total	76	100.00

**Table 2:** **Table 2:** Age distribution in study sample

*Groups*	*Age (years)*	*Frequency*
I	9-10	11
II	10-11	12
III	11-12	13
IV	12-13	18
V	13-14	22

**Table 3:** **Table 3:** Frequency of individual tooth fracture

*Tooth #*	*Frequency*	*Percentile*
11	56	52.336
12	4	3.738
21	42	39.25
22	11	10.28
31	1	0.93
32	1	0.93
41	1	0.93
42	1	0.93
		100.0

**Table 4:** **Table 4:** Frequency of tooth fracture and overjet

*Overjet*	*Groups*	*No. of patients*
0-2 mm	I	1
2-4 mm	II	8
>4 mm	III	67

**Table 5:** **Table 5:** Mean, standard deviation and t-test of each parameter used in the study

*Parameters*		*Mean ± SD*		*Z_cal_*		*Z_tab_*		*p-values*		*Significance*
Overjet		5.5855 ± 2.2021		14.195		1.96		< 0.05		S
SNA		79.7632 ± 2.2368		–8.17		1.96		< 0.05		S
SNB		76.0329 ±4.0121		–14.358		1.96		< 0.05		S
ANB		3.7303 ± 2.0776		7.260		1.96		< 0.05		S
1 to NA (angle)		26.7237 ±6.2814		6.556		1.96		< 0.05		S
1 to NA (linear)		6.3947 ± 2.2065		9.461		1.96		< 0.05		S
Interincisal angle		119.118 ±10.6395		–8.916		1.96		< 0.05		S

S: Significant

**Table 6a:** **Table 6A:** Mean, standard deviation and t-test of each parameter for male data

*Parameters*		*Male*		*Male (mean ± SD)*		*t_cal_*		*t_tab_ (74, 0.05)*		*p-value*		*Significance*
Overjet		46		5.6848 ± 1.8588		9.676		1.96		< 0.05		S
SNA angle		46		79.5217 ± 4.26627		–9.043		1.96		< 0.05		S
SNB angle		46		75.9348 ± 1.8062		–15.265		1.96		< 0.05		S
ANB angle		46		3.5870 ± 2.0064		5.364		1.96		< 0.05		S
Upper incisor to NA (angle)		46		26.7174 ± 6.0870		5.256		1.96		< 0.05		S
Upper incisor to NA (linear)		46		6.3152 ± 2.1842		7.189		1.96		< 0.05		S
Interincisal angle		46		118.2826 ± 11.3503		7.002		1.96		< 0.05		S

S: Significant

**Table 6b:** **Table 6B:** Mean, standard deviation and t-test of each parameter for female data

*Parameters*	*Female*	*Female (mean ± SD)*	*tcal*	*t_tab_ (74, 0.05)*	*p-value*	*Significance*
Overjet	30	5.4333 ± 1.4665	12.823	1.96	< 0.05	S
SNA angle	30	80.1333 ± 3.0369	–3.367	1.96	< 0.05	S
SNB angle	30	76.1833 ± 3.1472	–6.642	1.96	< 0.05	S
ANB angle	30	3.9500 ± 2.1985	4.858	1.96	< 0.05	S
Upper incisor to NA (angle)	30	26.7333 ± 6.6744	3.884	1.96	< 0.05	S
Upper incisor to NA (linear)	30	6.5167 ±2.2723	6.066	1.96	< 0.05	S
Interincisal angle	30	120.4000 ± 9.4890	–5.541	1.96	< 0.05	S

S: Significant

## DISCUSSION

Permanent anterior tooth fracture is a frequently encountered oral health problem. It causes a negative impact on the esthetics, speech, masticatory functions as well as psychology of both the patient and the parents thereby affecting the overall personality and daily life of an individual. Being a preventive dentist along with the pediatric dentist, it is our responsibility as well as our duty to opt for preventive measures rather than the cure and protect the child from unnecessary psychological trauma and hampered oral functions.

The proper knowledge of etiology and predisposing factors is necessary for early recognition and suitable treatment of patients who are at risk of permanent anterior tooth fracture. The present study shows that maxillary incisors are most commonly fractured tooth and as generally people are right handed, so the frequency of fractured right maxillary incisors were more than any other permanent tooth which is in agreement with the studies done by Baldava and Anup,^4^ Johnson^5^ and Ravn.^6^ Zuhal et al**^7^** have reported that the most affected age group was of 9 to 11 years old for sustaining permanent anterior tooth injuries and various other studies showed up to 12 years as more prone age group. So, we took an age group of 9 to 13 years old for our study and found that frequency of tooth fracture increased with increasing age. The possible reason being that with increasing age the child becomes more inquisitive and wants to explore new activities and areas untouched where chances of sustaining injury are more.

**Table 7a:** **Table 7A:** Mean, standard deviation and t-test of each parameter in male and female data combined

		*Sex*		*N*		*Mean*		*Std. deviation*		*Std. error of mean*
Overjet		Female		30		5.4333		1.4665		0.2677
		Male		46		5.6848		2.5828		0.3808
SNA		Female		30		80.1333		3.0369		0.5545
		Male		46		79.5217		1.8588		0.2741
SNB		Female		30		76.1833		3.1472		0.5746
		Male		46		75.9348		1.8062		0.2663
ANB		Female		30		3.9500		2.1985		0.4014
		Male		46		3.5870		2.0064		0.2958
Angle		Female		30		26.7333		6.6744		1.2186
		Male		46		26.7174		6.0870		0.8975
Linear		Female		30		6.5167		2.2723		0.4149
		Male		46		6.3152		2.1842		0.3220
Interincisal		Female		30		120.4000		9.4890		1.7324
		Male		46		118.2826		11.3503		1.6735

**Table 7b:** **Table 7B:** Unpaired t-test for each parameter for male and female data combined

	*t-test for equality of means*	*df*	*Significance (2-tailed)*	*Mean difference*	*Std. error difference*	*95% confidence interval of the difference*	
	*T*					*Lower*	*Upper*
Overjet	–0.484	74	0.630	–0.2514	0.5194	–1.2865	0.7836
	–0.540	72.859	0.591	–0.2514	0.4655	–1.1792	0.6763
SNA	1.090	74	0.279	0.6116	0.5610	–0.5063	1.7295
	0.989	43.238	0.328	0.6116	0.6185	–0.6355	1.8587
SNB	0.437	74	0.663	0.2486	0.5684	–0.8839	1.3810
	0.392	41.561	0.697	0.2486	0.6333	–1.0299	1.5270
ANB	0.742	74	0.460	0.3630	0.4890	–0.6113	1.3374
	0.728	58.026	0.469	0.3630	0.4986	–0.6351	1.3612
Angle	0.011	74	0.991	1.59402	1.4840	–2.9410	2.9729
	0.011	57.997	0.992	1.594E–02	1.5134	–3.0135	3.0453
Linear	0.387	74	0.700	0.2014	0.5208	–0.8362	1.2391
	0.384	60.355	0.703	0.2014	0.5252	–0.8490	1.2519
Interincisal	0.846	74	0.400	2.1174	2.5016	–2.8671	7.1019
	0.879	69.419	0.382	2.1174	2.4087	–2.6874	6.9222

**Table 8:** **Table 8:** Karl Pearson correlation coefficient for the overall data

				*Overjet*		*SNA*		*SNB*		*ANB*		*Angle*		*Linear*		*Interincisal*
Overjet		Pearson correlation		1.000		0.015		–0.332		0.402		0.214		0.291		–0.321
		Significance (2-tailed)		–		0.896		0.003		0.000		0.064		0.011		0.005
		N		76		76		76		76		76		76		76
SNA		Pearson correlation		0.015		1.000		0.626		0.427		–0.107		–0.198		0.108
		Significance (2-tailed)		0.896		–		0.000		0.000		0.356		0.087		0.353
		N		76		76		76		76		76		76		76
SNB		Pearson correlation		–0.332		0.626		1.000		–0.438		–0.017		–0.123		0.088
		Significance (2-tailed)		0.003		0.000		–		0.000		0.884		0.290		0.452
		N		76		76		76		76		76		76		76
ANB		Pearson correlation		0.402		0.427		–0.438		1.000		–0.104		–0.086		0.023
		Significance (2-tailed)		0.000		0.000		0.000		–		0.372		0.463		0.844
		N		76		76		76		76		76		76		76
Angle		Pearson correlation		0.214		–0.107		–0.017		–0.104		1.000		0.636		–0.475
		Significance (2-tailed)		0.064		0.356		0.884		0.372		–		0.000		0.000
		N		76		76		76		76		76		76		76
Linear		Pearson correlation		0.291		–0.198		–0.123		–0.086		0.636		1.000		–0.369
		Significance (2-tailed)		0.011		0.087		0.290		0.463		0.000		–		0.001
		N		76		76		76		76		76		76		76
Interincisal		Pearson correlation		–0.321		0.108		0.088		0.023		–0.475		–0.369		1.000
		Significance (2-tailed)		0.005		0.353		0.452		0.844		0.000		0.001		–
		N		76		76		76		76		76		76		76

Males are more prone to tooth fractures than females.^8,9^It may be due to their aggressive and energetic nature. The present study shows that with increasing overjet, frequency of tooth fracture also increased in agreement with other studies by Grimm et al.^10^

Hamdan et al^11^ says that children with overjet greater than 5 mm sustained significantly more injuries to incisor teeth than children with normal overjet. But, one such study done by Stokes, Loh^11^ found that the incisal overjet is not a positive correlate with traumatic dental injury in Singapore children.

Less number of studies has been done to establish relationship between permanent anterior tooth fracture and craniofacial morphology. In the present study, we have correlated anterior tooth fracture with following parameters *viz* occlusion, overjet, SNA angle, SNB angle, ANB angle, interincisal angle, upper incisor to NA (both linear and angular measurement).

In our study, we have also taken various skeletal and dental parameters to get a more accurate idea of the various craniofacial morphological factors predisposing a person to permanent anterior tooth fracture. So, our study helps in better assessment of the patients who are at risk of having permanent anterior tooth fracture. At the same time the conclusions drawn from the study are also of help in treating orthodontic patients as we are coming to know the relationship of various parameters with permanent anterior tooth fracture like SNA angle, SNB angle, interincisal angle, etc.

But at the same time, we should not forget that the growth is not complete at this age and the patients who seem to be having class II malocclusion might develop a normal class I occlusion. So, skeletal parameters are not very much predictive of permanent anterior tooth fracture. In addition, anterior tooth proclination is important in predicting the likelihood of getting a tooth fractured. So, it is the dental parameter, i.e. the increased overjet which is more responsible for anterior tooth fracture.

Through the knowledge of the correlation we can understand about the type of effect a parameter will have on the other craniofacial components which is in long run very helpful in treating the orthodontic patients at risk of permanent anterior tooth fracture. But more work is needed on the observations and results made from the present study before these results can be applied for clinical application and treatment of orthodontic patients.
